# Habitat Management to Reduce Human Exposure to *Trypanosoma cruzi* and Western Conenose Bugs (*Triatoma protracta*)

**DOI:** 10.1007/s10393-016-1153-5

**Published:** 2016-08-11

**Authors:** Lisa Shender, Michael Niemela, Patricia Conrad, Tracey Goldstein, Jonna Mazet

**Affiliations:** 1 One Health Institute, School of Veterinary Medicine, University of California, Davis, Davis, CA 95616 USA; 2 California Department of Public Health, Sacramento, CA USA

**Keywords:** *Trypanosoma cruzi*, Chagas disease, *Triatoma*, *Neotoma*, California, Microhabitat

## Abstract

Chagas disease, which manifests as cardiomyopathy and severe gastrointestinal dysfunction, is caused by *Trypanosoma* *cruzi*, a vector-borne parasite. In California, the vector *Triatoma* *protracta* frequently colonizes woodrat (*Neotoma* spp.) lodges, but may also invade nearby residences, feeding upon humans and creating the dual risk of bite-induced anaphylaxis and *T*. *cruzi* transmission. Our research aimed to assess *T*. *cruzi* presence in woodrats in a previously unstudied northern California area, statistically evaluate woodrat microhabitat use with respect to vegetation parameters, and provide guidance for habitat modifications to mitigate public health risks associated with *Tr*. *protracta* exposure. Blood samples from big-eared woodrats (*N*. *macrotis*) trapped on rural private properties yielded a *T*. *cruzi* prevalence of 14.3%. Microhabitat analyses suggest that modifying vegetation to reduce understory density within a 40 meter radius of human residences might minimize woodrat lodge construction within this buffer area, potentially decreasing human exposure to *Tr*. *protracta*.

**Electronic supplementary material:**

The online version of this article (doi:10.1007/s10393-016-1153-5) contains supplementary material, which is available to authorized users.

## Introduction

In the United States, at least 24 wildlife species are documented hosts for *Trypanosoma* *cruzi* (Bern et al. 2011), a zoonotic vector-borne parasite that causes Chagas disease in humans and dogs. Characterized by chronic cardiomyopathy and severe gastrointestinal dysfunction, Chagas disease has an insidious onset in humans. Clinical symptoms generally do not present until decades after vectorial pathogen transmission has occurred, at which point the patient may have missed the window of opportunity for effective chemotherapeutic treatment of this often fatal disease (Marin-Neto et al. 2009). Although Chagas disease is most frequently acquired in Latin America, locally acquired cases are periodically documented in the United States (Bern et al. 2011), and recent serological studies indicate that local pathogen exposure may occur more frequently than previously realized (Cantey et al. 2012). Therefore, where the risk of Chagas disease transmission exists, it is important to improve upon regional knowledge of *T*. *cruzi* reservoirs and vectors, especially in less-studied regions, such as parts of northern California.

In California, western conenose bugs (*Triatoma* *protracta*) are often infected with *T*. *cruzi*, as was first discovered in 1916 from specimens collected within a woodrat’s large stick house, or lodge, in San Diego County (Kofoid and Donat 1933). The close ecological association between woodrats (*Neotoma* spp.) and conenose bugs creates ideal conditions for a sylvatic *T*. *cruzi* transmission cycle, with woodrats serving as a primary reservoir. Furthermore, several woodrat species can adapt to peridomestic environments, nesting in and around private property structures located within woodrat habitat, resulting in an interface where *T*. *cruzi* can spillover from the sylvatic cycle to domestic animals and humans. When zoonotic pathogens, such as *T*. *cruzi*, are present in the local rodent population, it is prudent to practice control measures to reduce the risk of disease transmission to rural residents. However, the use of rodenticides, a seemingly attractive quick-fix for rodent control, is impractical at the landscape level and potentially damaging to local populations of nontargeted wildlife species, due to direct or secondary toxicity effects (Stone et al. 1999; Riley et al. 2007). In contrast, habitat modification may serve as a nontoxic means of managing woodrat populations, yet methods to implement this option have not been well described or made publicly available to property owners.

Although woodrat habitat use in California has been studied in relatively pristine woodland areas, the use of microhabitats has not been examined on private properties where *T. cruzi* transmission is a risk and anthropogenic factors might affect woodrat behavior. Dense vegetation supports larger woodrat populations and construction of woodrat lodges (Fargo and Laudenslayer 1999), thus providing more sites for conenose bug colonies. During the warm summer months, adult bugs disperse from their colonies and, when drawn to nearby lights, can invade human residences. Once in the home, the bugs find refuge in furniture (e.g., beds and couches) and animal bedding to emerge nightly and feed upon people and their pets. In addition to posing a risk for Chagas disease transmission, the bite of *Tr*. *protracta* can be allergenic and incite severe anaphylaxis (Moffitt et al. 2003; Klotz et al. 2010). The annual incidence of allergic reactions to *Tr*. *protracta* bites is unknown (Moffitt et al. 2003). However, the finding that 6.7% of study participants had elevated *Tr*. *protracta*-specific IgE antibodies led authors to conclude that 30,000 Californians were susceptible to bite-induced allergic reactions (based on 1983 California population data) (Marshall et al. 1986). Thus, it is clear that preventive steps to reduce home invasion by conenose bugs should be followed in high-risk areas.

Public information is lacking on how small-scale microhabitat characteristics and alterations influence the presence or absence of vectors and reservoirs of *T. cruzi* near rural home dwellings. Furthermore, the prevalence of *T*. *cruzi* in northern California woodrat populations has not been studied. Therefore, our primary research goals were to (1) assess a northern California woodrat population for the presence of *T. cruzi*; and (2) evaluate woodrat capture locations with respect to vegetation density in the rural home environment. In addition, combining our data with previous studies on woodrat microhabitat use in California, we aimed to propose habitat modifications that should minimize woodrat activity adjacent to human dwellings, thereby potentially reducing human exposure to conenose bugs and *T*. *cruzi*. Our study is unique in that it was performed on parcels of rural private property, and we have integrated data on woodrat habitat use and *T*. *cruzi* infection. Our suggestions serve as a platform for future studies designed to test woodrat response to microhabitat modifications on residential parcels. Meanwhile, until such studies have been accomplished, when providing advice to people faced with the dual risk of *T*. *cruzi* transmission and severe conenose bug allergies, the public health sector should consider our proposed habitat modifications, in addition to standard rodent exclusion techniques, as a likely means of woodrat control.

## Materials and Methods

### Field Methods

#### Rodent Trapping and Sample Collection

Woodrats were trapped on 4 private properties in Vallecito (38.0903°N, 120.4736°W), located in the foothills of Calaveras County, from June to October 2012. Conenose bugs collected from one of these properties tested positive for *T*. *cruzi* a year before our study’s initiation (M. Niemela, unpublished data), justifying study site selection. Trapping grids were established on each property with the size and orientation constrained by topography and property boundaries. Grids consisted of 5–7 parallel line transects spaced approximately 10 m apart with trap stations located at roughly 10-m intervals along each transect length. Two traps (Sherman or Tomahawk model #201) were placed within a 2 m radius of each station, based on microhabitat features that improved the probability of rodent capture. Exposed traps were covered with twigs and vegetation to provide protection from environmental elements, and all traps included cotton balls as insulation. Traps were baited with a mixture of oats, peanut butter, and sunflower seeds no earlier than 90 min before sunset and were checked within one hour of sunrise for 4 consecutive nights/trapping session. Trapping was performed twice on each property at approximately 2-month intervals and was conducted in compliance with the California Department of Fish and Wildlife (permit #SC-003492) and the University of California, Davis Animal Use and Care Administrative Advisory Committee (protocol #16816). Rodent handling incorporated recommended safety protocols, including the use of appropriate personal protective equipment (i.e., nitrile gloves, protective eye wear, N95 mask, and Tyvek suit).

Captured woodrats were transferred to a sturdy pillowcase and weighed prior to being anesthetized within a 3.5-L glass jar using a 50:50 mixture of isoflurane/propylene glycol at a dosage of 1 mL/500 mL container volume (Itah et al. 2004). The anesthetic solution was applied to 3 absorbent gauze pads (4” nonwoven 4 ply) inserted into a mesh wire basket affixed beneath the jar’s lid, to prevent rodents from directly contacting the solution. To maintain appropriate anesthetic depth during blood collection, additional anesthesia was provided using a 6 cc syringe case as an inhalation nose-cone. A conservative quantity of blood (<10% total blood volume, where total blood volume was calculated as 6% body mass; Mitchell 2011) was collected into 500-µL EDTA Microtainer® tubes from the retro-orbital plexus via heparinized capillary tubes. Application of monel numbered ear tags (#1005-1, National Band and Tag Company) facilitated individual identification across trapping sessions. After processing, woodrats were monitored within their traps until alert and ready for release at their original capture location. Individual rodents were bled once during each 4-day capture session (i.e., blood not taken from recaptures). Blood samples were stored in a cooler on ice until they could be centrifuged in the field (<12 h). Following centrifugation, the plasma was removed without disturbing the buffy coat layer. The remaining whole blood (thin layer of plasma, buffy coat, and red blood cells) was homogenized and aliquoted into microcentrifuge tubes for laboratory DNA extraction and *T*. *cruzi* PCR assays. The survival study and requisite small quantity of blood collected precluded *T*. *cruzi* detection via hemoculture, without which serology would be uninformative due to cross-reactions with other trypanosomes commonly harbored by woodrats (Charles et al. 2012; Upton et al. 1989; Wood and Wood 1937).

#### Vegetation Microhabitat Analyses

A field tape measure was used to define a 3-m radius circle, centered at each trapping station. Each circle was quartered, and the percentages of total understory and overhead canopy, as well as common plant species, were visually approximated and recorded. Understory consisted of downed wood and vegetation (excluding tall grasses) ≤1 m tall and canopy of vegetation ≥3 m. All observations were made by the same field researcher to maintain consistency in methodology.

To assess woodrat habitat use preferences at a microhabitat level, we used mixed-model logistic regression analyses to evaluate the potential association between vegetation cover and woodrat capture success, as defined by a woodrat ever being captured at a trap location. Study site was included as a random effect variable with a varying intercept. The effects of canopy and understory were initially assessed via univariate analysis of their continuous percent values. For ease of model interpretation, and consistent with previous research (Cranford 1977), models were repeated using categorical data, whereby the continuous percent values of significant vegetation variables were converted into 4 factor levels: 0–25%, >25–50%, >50–75%, and >75–100%. Estimate biases related to potential predictor and unit effect correlations (i.e., vegetation structure correlated with study site) were corrected for by including variables that represented the average percent canopy and understory cover by study site (Bafumi and Gelman 2006). All models were run in R using the lme4 package (R Development Core Team 2011).

### Laboratory Methods

The DNeasy Blood and Tissue or QiaAmp DNA Blood Mini kit (Qiagen; Germantown, MD) was used to extract DNA from 100–200 µL of whole blood. For each sample, DNA quality was evaluated via quantification of nucleic acid concentration (µQuant/Gen5 data analysis software, BioTek Instruments Incorporated) and the presence of the interphotoreceptor retinoid-binding protein (IRBP) gene via PCR (Ferreira et al. 2010). *Trypanosoma cruzi* DNA was detected using 2 conventional PCR assays with previously published primers TcZ1/TcZ2 (Moser et al. 1989) and 121/122 (Wincker et al. 1994). For both assays, DNA from the *T*. *cruzi* Y-strain (kindly provided by the CDC Division of Parasitic Diseases) was used as a positive control. The TcZ1/TcZ2 PCR products from 3 positive woodrats were cloned (TOPO® TA Cloning Kit; Invitrogen Cat#: K4575-01; Life Technologies, Carlsbad, CA) (Woodman 2008) and sequenced at the UC Davis Sequencing Lab (ABI Prism analyzer and software). The resulting sequences were trimmed (Geneious version 5.3.6, http://www.geneious.com) to remove the TcZ1 and TcZ2 primers and compared with sequences in the GenBank database to confirm the presence of *T*. *cruzi* DNA. To assess the potential of PCR inhibitors, for a subset of negative samples, 0.96 mg/mL of bovine serum albumin was added to the PCR buffer (Chen et al. 2007) or diluted (1:10) DNA template was added to the PCR reaction and tested as above. The reaction and cycling conditions for all assays are provided in Table S1.

## Results

### Property Descriptions

The habitat type across all properties was a combination of chaparral and blue oak-foothill pine (Mintier and Associates and ESA Associates 2008) with dominant plant species as listed in Table 1. Property owners’ personal preferences in land management resulted in a diversity of vegetation structure and composition among sites (Table 1). At Site 1, a 20–25-m-wide swath of cleared land bisected the trapping grid horizontally into upper and lower sections. Sites 3 and 4 shared a fence line but differed greatly in vegetation structure. The trapping grids on these 2 properties were separated by more than 40 m, allowing us to evaluate these sites as separate entities.

**Table 1:** Trapping Grid Characteristics and Woodrat Captures and Lodges by Study site in Vallecito, Calaveras County, California.

Site^a^	WRs^b^	Lodges^c^	Understory^d^	Canopy^d^	Maintenance^e^	Structures^f^	Trapping grid description
1	12 (3)	10 (3)	47.0 (50)	55.8 (55)	None	No	Densely vegetated with the exception of a 20-meter wide grassy swath completely void of understory and canopy where no WRs were trapped
2	5	2	24.2 (22)	77.5 (90)	Moderate	Yes	Grassy open fields interspersed among oak and pine trees on an eastward facing slope; ornamental hedge by rock wall
3	16 (5)	18 (4)	51.4 (55)	77.1 (95)	None	No	Undeveloped land leased for cattle grazing; large-girth oak trees with expansive secondary branching of trunk base; dense berry vine patches; abundant downed wood (i.e., downed trees and smaller woody debris); grassy areas free from shrubbery, likely from cattle impact, scattered among oak trees
4^g^	7 (1)	1 (1)	21.7 (15)	77.6 (92)	High	Yes	Fruit trees adjacent to trapping grid; ornamental, mat-forming nonnative groundcover (*Vinca* spp.) where no WRs were trapped

^a^Across all sites, dominant native tree species: interior-live oak (*Quercus* *wislizenii*), blue oak (*Q. douglasii*), and gray pine (*Pinus sabiniana*). Common understory species: toyon (*Heteromeles arbutifolia*), redberry (*Rhamnus crocea*), manzanita (*Arctostaphylos* spp.), poison oak (*Toxicodendron diversilobum*), chamise (*Adenostoma fasciculatum*), and buckbrush (*Ceanothus* *cuneatus*).

^b^Number of woodrats captured per trapping grid (number of WRs captured at targeted dens on property but off the trapping grid).

^c^Number of woodrat lodges detected within the trapping grid (additional lodges outside of the grid at which traps were placed).

^d^Mean (median) percent understory and canopy cover based on trapping station measurements.

^e^Moderate = periodic control of poison oak and some clearing of downed brush; High = carefully tended with complete clearing of brush piles and trimming of lower branches of oak trees.

^f^Site 2: workshed, abandoned chicken coop, and a small rock wall near the owner’s home; Site 4: well pump station, drainage pipe culvert, small guest house.

^g^Several WR carcasses were found on the north edge of the trapping grid during the August trapping session, and it was discovered that a neighbor was using rodenticide in a shed close to the property fence line.

### Trapping Results

Across all trapping grids and sessions (2250 trap nights), there were a total of 89 woodrat captures, representing 40 individual woodrats. Nine additional woodrats were captured at targeted lodges beyond the trapping grid boundaries. Table 1 provides details on woodrat captures by site. Of the 18 woodrats captured at multiple trap stations, the greatest travel distance was 30.5 m with an average distance of 18.6 m. There was no overlap between woodrats captured on the upper and lower portions of the grid at Site 1, indicating that woodrats likely avoided moving across the bare swath of ground dividing the grid. Similarly, there was no overlap in individual woodrats captured at adjacent Sites 3 and 4.

### Microhabitat

Woodrat captures were significantly associated with understory but not with canopy cover (Table 2). There was a striking 24-fold increase in the odds of a woodrat being captured at a trap station with understory coverage of >75–100% as compared to the reference category of 0–25%.

**Table 2:** Vegetation Cover as Predictors of Woodrat Capture Success at Individual Trap Stations (*n* = 164), Based on Mixed-Model Logistic Regression with Study Site as a Random Effect Variable.

Variable^a^	Factor level (%)	OR^b^	95% CI^c^	AIC^d^
Continuous understory	n/a	1.05	1.027–1.063	149.38
Continuous canopy	n/a	1.01	0.998–1.022	179.33
Categorical understory	0–25	Ref	n/a	150.86
	>25–50	3.2	1.1–9.1	
	>50–75	9.8	3.1–30.8	
	>75–100	24.3	5.2–112.7	

^a^Understory defined as downed wood and vegetation (excluding grasses) ≤1 m; Canopy defined as vegetation ≥3 m.

^b^Odds ratios. For continuous variable models, the ORs indicate the increased likelihood of woodrat capture success for each 1% increase in canopy or understory coverage. For the categorical model, the ORs represent the increase in odds as compared to the reference category of 0–25% understory.

^c^Intervals that do not contain the value 1.0 indicate variable significance.

^d^Akaike information criterion. The lowest AIC value indicates an improved model fit for the evaluation of comparable models.

### Laboratory Results

Seven of the 49 (14.3%) individual woodrats tested positive for *T*. *cruzi* (6 via the nuclear TcZ1/TcZ2 and 7 via the kinetoplast 121/122 assay). Notably, of the 2 positive woodrats that were captured across trapping sessions, both were positive in August but negative when recaptured in October. An IRBP band was visible for all samples indicating that we obtained good quality DNA. The use of BSA (*n* = 17) or diluted DNA template (*n* = 12) did not alter the negative outcome of the initial PCR assays. Nuclear DNA sequences from 3 positive woodrats (GenBank accession numbers KM657483–KM657485) confirmed the presence of *T*. *cruzi* with up to 99% sequence identity (GenBank accession number HM015642).

## Discussion

### *T. cruzi* Findings

Reported research on *T*. *cruzi* in northern CA wildlife is nonexistent, with one exception. In 1982, a locally acquired human case of Chagas disease occurred in Tuolumne County, about 65 km south of our study area. During follow-up investigations, 28 rodents were tested for *T*. *cruzi* (Navin et al. 1985). Five woodrats and 4 deer mice (*Peromyscus* *maniculatus*) were negative, but 2 of 19 ground squirrels (*Otospermophilus* *beecheyi* ***)*** tested positive. Our study identified woodrats as *T*. *cruzi* reservoirs (14% of 49 woodrats tested positive) in the foothills of Calaveras County, northern California. In complementary research, Shender et al. (2016) performed genotyping and phylogenetic analyses of *T*. *cruzi* detected in *Tr*. *protracta* specimens obtained from the same private properties on which we conducted our rodent trapping. In fact, some of the *T*. *cruzi* positive vectors were collected directly from excavated woodrat lodges that housed positive woodrats in this study. Although the virulence of local *T*. *cruzi* strain(s) is completely unknown and although proven cases of local transmission have been exceedingly rare (Bern et al. 2011), rural residents should be cognizant of the potential risk of Chagas disease associated with these rodents and the conenose bugs that inhabit their nests.

Although a *T*. *cruzi* prevalence of 14% in *N*. *macrotis* is not inconsequential, it is lower than previously reported for southern plains woodrats (*N*. *micropus*) in Texas (Pinto et al. 2010; Charles et al. 2012). However, we suspect that the true prevalence in our study area could be higher, since 2 of the woodrats were positive when first captured, but negative upon recapture 2 months later. *Trypanosoma* *cruzi* is presumed a life-long infection, not cleared by the host’s immune system (Yabsley et al. 2001). It is therefore probable that these 2 woodrats were initially sampled during the acute phase of infection, when parasitemia levels were high enough for efficient PCR detection of *T*. *cruzi* DNA in peripherally collected blood samples. The second sampling of these animals probably occurred during the chronic phase, characterized by parasite pseudocysts in cardiac muscles and other tissues and a relatively low level of parasitemia (Yabsley et al. 2001), thereby reducing the efficiency of *T*. *cruzi* PCR detection. This finding suggests that other woodrats sampled in our study area also likely had chronic infections, and thus escaped detection via our sampling methods. This reasoning is supported by research in Texas, in which sampling protocols included rodent euthanasia, allowing for collection of maximum quantities of blood and the performance of multiple diagnostic assays (Charles et al. 2013). These authors found that the overall *T*. *cruzi* prevalence in *N*. *micropus* was 66% based on a combination of 4 diagnostic techniques (PCR, serology, blood smears, and culture), but only 22% when PCR alone was considered (Charles et al. 2012). Our priority was to obtain a cross-sectional prevalence of woodrats with circulating *T*. *cruzi* organisms (i.e., the percent of the woodrat population that could efficiently transmit *T*. *cruzi* to uninfected conenose bug vectors). Therefore, PCR of blood samples was considered to be the optimal diagnostic assay for our study, in which live-capture methods necessitated collection of smaller quantities of blood than would have been possible had we followed a euthanasia protocol.

### Management Recommendations

The presence of *T*. *cruzi* in big-eared woodrats and the potential for pathogen transmission to humans and domestic pets underscore the necessity for mitigation of human exposure to both woodrats and conenose bugs. Prevention strategies to decrease in-home exposure to western conenose bugs (e.g., minimizing outdoor lighting, closing curtains in lighted rooms during times of bug dispersal) are available for those who are knowledgeable about the risks and aggressively seek them out (California Department of Public Health Division of Communicable Disease Control 2010). Targeted control of woodrats has focused on methods to exclude these rodents from the home, such as sealing potential entry points to protected nesting environments (i.e., attics, crawl spaces, etc.) with rodent-proof material (California Department of Food and Agriculture 2009). The results of our research suggest that upstream control via careful vegetation management could serve as an additional means to discourage woodrats from constructing lodges in and around human residences.

In our study region, *N*. *macrotis* preferentially use microhabitats with densely vegetated understory. A dense understory promotes habitat use and favors woodrat survival by offering woodrats shelter from predators and providing sturdy scaffolding upon which to construct their lodges (Cranford 1977; Sakai and Noon 1997). Downed pieces of wood (e.g., fallen branches and chunks of tree bark) are used both as building material and as on-the-ground “quiet roadways” to minimize noise created when moving through leaf litter and decrease predator auditory detection (Innes et al. 2007).

As opposed to understory density, canopy cover was not significantly associated with woodrat capture success in our study. In contrast, some other studies have found canopy to be significantly associated with woodrat activity and lodge location (Gerber et al. 2003), demonstrating that in some regions, canopy cover might be equally important to woodrat survival. A thick overhead canopy provides woodrats visual protection from aerial predators, an escape route from terrestrial predators, and corridors to move across patchy areas of habitat with little understory growth (Laudenslayer and Fargo 1997). However, as it appears that regional differences exist with respect to the importance of canopy cover, understory modification should be most strongly considered in management practices.

Our study was unique in that it was conducted on private property with anthropogenic influences, such as the presence of ornamental plants, livestock, domestic pets, and human structures. Therefore, a valuable outcome was the discovery that woodrat habitat use on rural private properties was similar to the habitat use in more pristine (Cranford 1977; Sakai and Noon 1997) areas. As this study and others have shown, woodrats tend to avoid open habitat patches where the rodents are more prone to predation (Cranford 1977; Sakai and Noon 1997). For example, Cranford (1977) determined that woodrats seldom used areas of 0–25% vegetation cover and that woodrat biological centers of activity were always within habitat containing ≥50% vegetative cover.

During our 4-day trapping period, the maximum movement distance of an individual woodrat was approximately 30 m. Similarly, 32 m was the greatest movement distance observed for any individual woodrat captured from the same nest in rural canyons of San Diego County, California (Smith 1965). The woodrat movement data observed, as well as statistical habitat analyses performed in this and previous research, provide quantitative data to guide vegetation modifications aimed at reducing woodrat activity around human homes. We therefore conjecture that data from our study and others may be applied by residents to either exclude woodrats from, or direct their presence to, particular areas of the rural property. For example, landscape might be managed to create islands of suitable (i.e., woody understory) or unsuitable (e.g., open areas such as grasses, flower and vegetable gardens, and patios) habitat. Specifically, landscape features such as stumps and logs can favor woodrat lodge construction, while rocks, bare ground, and mat-forming shrubs may be negatively associated with woodrat lodge presence (Innes et al. 2007).

However, we do not condone the complete elimination of woodrats, and the destruction of all woodrat lodges on rural property is inadvisable for several reasons. First, adult *Triatoma* spp. dispersal is strongly hunger driven (Sjogren and Ryckman 1966; Ekkens 1981; Lehane and Schofield 1982), so complete rodent removal could force conenose bugs to seek alternative blood sources, placing humans more at risk for allergic reactions from bites and disease transmission (Cordell and Baker 1999). Secondly, the presence of woodrats provides many ecosystem benefits. The lodges offer shelter and feeding grounds for multiple wildlife taxa, including reptiles, amphibians, and birds, as well as other small mammals (Wood 1934; Vestal 1938), and the woodrats themselves are prey for numerous predator species (Sakai and Noon 1997; Gerber et al. 2003). Because woodrats play an important role in community dynamics and increase species biodiversity (Innes et al. 2007), we contend that conditions should be created to promote harmonious coexistence with these rodents, while minimizing the risk of human exposure to *T*. *cruzi* and conenose bugs. Genetic research on local *T*. *cruzi* strains is needed to provide insight on their relationship to those strains found in Latin America known to cause human disease. Until the potential virulence of California *T*. *cruzi* strains is better understood, property owners should work to modify their landscape to balance woodrat population control with natural ecosystem processes.

## Conclusion

This study complements simultaneous research by Shender et al. (2016) on *T*. *cruzi* in the vector *Tr*. *protracta*, and demonstrates for the first time that the Chagas disease-causing pathogen is harbored by woodrats in California as far north as Calaveras County. In addition, our data combined with those from previous studies, indicate that landscape vegetation management at the individual property level might influence the pattern of woodrat peri-urban habitat use. Thus, we believe that data-supported landscape management tools should be considered by public health representatives who provide written and verbal advice regarding methods to discourage woodrats from building lodges near human homes in California foothill counties. The careful application of concentric buffers around human residences, in combination with typical rodent exclusion methods, might greatly reduce woodrat activity adjacent to human homes.

**Figure 1:**
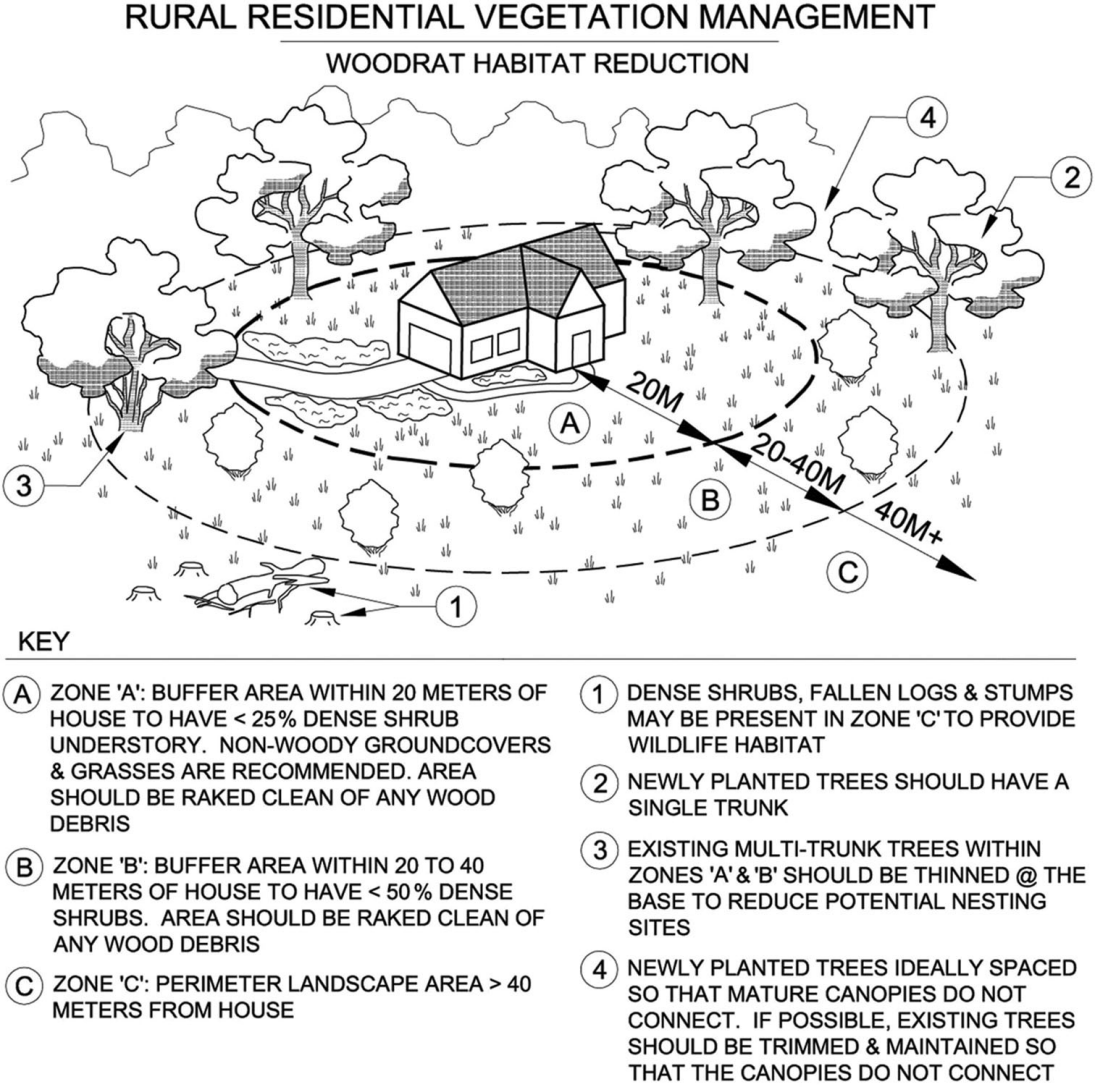
Suggested guidelines for habitat modifications to deter big-eared woodrats (*Neotoma macrotis*) from constructing lodges near human homes located on rural properties in the foothills of northern California.

Residents who live on large rural properties with an abundant woodrat population, and who seek additional methods to control the activity of these rodents, might try surrounding the home with a patchy gradient of vegetation (see Fig. 1). Specifically, we suggest that within the first 20 m of the home, the density of shrubby understory be less than 25%. Ideally, vegetation in this first buffer ring would mainly consist of nonwoody plants, such as mat-forming groundcover and grasses. A second buffer ring should extend 20–40 m from the home, with no more than 50% ground coverage of woody vegetation. Large tree stumps and downed logs should be removed from the designated buffer areas. Shade trees, if present, should preferably be species possessing a single trunk (i.e., without low-level secondary branching or forked bases) and be spaced to avoid forming a connecting canopy. The implementation of these habitat modifications, which partially align with California fire prevention laws for the creation of a “defensible space” (California Department of Forestry and Fire Protection CAL FIRE 2007), could create a zone of suboptimal habitat for woodrats. The proposed habitat modifications should result in decreased woodrat activity and an absence of (or far fewer) woodrat lodges in close proximity to human homes. Despite the ability of triatomine bugs to disperse >40 m, eliminating woodrat lodges within this buffer zone, along with reducing outdoor lighting during times of bug dispersal, should greatly decrease human exposure to the western conenose bug (*Tr*. *protracta*) and the accompanying risk of anaphylaxis and *T*. *cruzi* transmission.

## Electronic supplementary material

Below is the link to the electronic supplementary material.

Supplementary material 1 (DOCX 14 kb)
