# Supplementary material for: Habitat Management to Reduce Human Exposure to Trypanosoma cruzi and Western Conenose Bugs (Triatoma protracta)
Source: Ecohealth. 2016 Aug 11;13(3):525–34. doi: 10.1007/s10393-016-1153-5 (PMC5063897; doi:10.1007/s10393-016-1153-5)
Supplement: Supplementary file 1 — Supplementary material 1 (DOCX 14 kb) [file 10393_2016_1153_MOESM1_ESM.docx]

**Table S1:** PCR assays for *T*. *cruzi* screening and interphotoreceptor retinoid-binding protein (IRBP) gene.

| **PCR Assay** | **Forward/Reverse primers (Reaction Conditions)** | **Cycling conditions (’ = minutes; ” = seconds)** | **bp** |
| --- | --- | --- | --- |
| nuclear 195bp satellite | TcZ1: 5'-CGA GCT CTT GCC CAC ACG GGT GCT-3' / TcZ2: 5'-CCT CCA AGC AGC GGA TAG TTC AGG-3' (20µl: 1.9mM MgCl_2_, 0.2mM each dNTPs, 0.5µM each primer, 0.25U Taq & 4µl 5X buffer) | 94° x 5’; (94° x 20”, 57° x 10”, 72° x 30”)^35^; 72° x 7’ | 188 |
| kDNA minicircle | 121: 5'-AAA TAA TGT ACG GGK GAG ATG CAT GA-3' / 122: 5'-GGT TCG ATT GGG GTT GGT GTA ATA TA-3' (25µl: 1.5mM MgCl_2_, 0.2mM each dNTPs, 0.4µM each primer, 0.625U Taq & 5µl 5X buffer) | 95° x 10’; (94° x 30”, 58° x 30”, 72° x 1’)^35^; 72° x 10’ | 330 |
| IRBP | For: 5’-TCC AAC ACC ACC ACT GAG ATC TGG AC-3’ / Rev: 5’-GTG AGG AAG AAA TCG GAC TGG CC-3’ (25 µl: 1.5mM MgCl2, 0.4mM each dNTPs, 0.5µM each primer, 0.75U Taq, & 2.5 µl 10X buffer) | 94° x 4’; (94° x 30”, 57° x 30”, 72° x 1’)^35^; 72° x 5’ | 227 |
